# Network Localization of Anxiety as an Early Manifestation of Alzheimer’s Disease-related Neurodegeneration

**DOI:** 10.64898/2026.10.24.761536

**Published:** 2026-10-26

**Authors:** Soyoung Lee, Junghee Ha, Arun H. Garimella, Sheena R. Baratono, Grace T. Burt, Helmet T. Karim, Carmen Andreescu, Reisa Sperling, Nancy Donovan, Michael Fox, Shan Siddiqi

## Abstract

**Objective:**

Anxiety is a prominent neuropsychiatric symptom in preclinical Alzheimer’s disease (AD), but the neural circuitry linking anxiety and AD remains uncharacterized. We assessed whether anxiety in preclinical AD is independently associated with AD-related neurodegeneration and localizes to a common anxiety network.

**Methods:**

In cognitively unimpaired (CU) older adults with 3T MRI and amyloid-β PET (N=1,775), we examined whether an established AD-related atrophy network is associated with anxiety (STAI-6). Then, using Atrophy Network Mapping (ANM), which maps individual cortical atrophy patterns onto a normative functional connectome (N=1000), we derived an anxiety network. We tested its 1) spatial convergence with the AD network and with a causal anxiety network derived from lesion and neuromodulation data, 2) specificity relative to depression, 3) generalizability in samples from independent cohorts with AD and Parkinson’s disease (total n=878), and 4) association with longitudinal outcomes.

**Results:**

In CU older adults, greater AD-network atrophy was associated with higher anxiety, independent of depression, cognition, cortical atrophy, and amyloid burden. Anxiety localized to a network closely resembling the AD network (r=0.74, p=0.04) and the causal anxiety network (r=0.72, p=0.02). This convergence extended across the AD continuum and to the Parkinson’s disease cohort and was specific to anxiety relative to depression. Greater anxiety-network atrophy was associated with cognitive decline over 4.6 years.

**Conclusion:**

Anxiety localizes to a brain circuit resembling the AD-related atrophy network, is consistent across the AD continuum and may extend beyond AD. These findings support anxiety as an independent marker of early AD-related neurodegeneration.

## Full Text Availability

The license terms selected by the author(s) for this preprint version do not permit archiving in PMC. The full text is available from the preprint server.

